# Traditional Serrated Adenoma of the Gallbladder, a Case Report

**DOI:** 10.3389/pore.2022.1610133

**Published:** 2022-02-04

**Authors:** Tamás Micsik, Anna Jakab, Csaba Lehoczki, Árpád V. Patai

**Affiliations:** ^1^ St. Department of Pathology and Experimental Cancer Research, Semmelweis University, Budapest, Hungary; ^2^ Pathology Department, St. George University Teaching Hospital at Fejér County, Székesfehérvár, Hungary; ^3^ Interdisciplinary Gastroenterology Working Group, Semmelweis University, Budapest, Hungary; ^4^ Surgery Department of Bajcsy-Zsilinszky Hospital, Budapest, Hungary; ^5^ Department of Surgery, Transplantation and Gastroenterology, Semmelweis University, Budapest, Hungary

**Keywords:** traditional serrated adenoma, gallbladder, dysplasia, serrated-pathway, TSA

## Abstract

While overwhelming majority of laparoscopic cholecystectomy specimens performed for gallstones or cholecystitis show rather typical findings, sometimes polypoid structures are also removed. These can be related to cholesterolosis or conventional adenomas, but occasionally extraordinary findings do emerge. In our case, a 67-year old lady with typical complaints of cholecystitis underwent routine laparoscopic cholecystectomy. Preoperative ultrasound revealed a polypoid mass with inflammation and without suspicion for malignancy. Microscopic examination showed partly conventional, low-grade dysplastic crypts forming a villous and rather complex structure. Ectopic crypt foci, slit-like serration pattern and serrated dysplasia with eosinophylic cytoplasm and centrally located nuclei were seen throughout the lesion, thus a traditional serrated adenoma (TSA) of the gallbladder was diagnosed. TSA represents the rarest subtype of serrated lesions in the colon and extracolonic manifestations are sporadically reported. Until now only a single case of a serrated adenoma was reported from the gallbladder. Here we describe the detailed clinical, pathological and molecular findings of our case and discuss these in the light of current literature data regarding this field.

## Introduction

Gallbladder specimens removed due to cholecystitis represent a large segment of surgical specimens with few remarkable changes of which carcinoma has the highest importance [1]. According to clinical guidelines polypoid lesions (∼3%) of the gallbladder should be removed if larger than 10 mm or rapidly growing [2]. Adenoma is relatively rare (0.1–1%) [3] and usually develops in inflamed mucosa of adult females as tubulary or villous structures, sometimes in association with familial adenomatous polyposis (FAP) or other polyposis syndromes [4]. There are a few case reports of larger adenomas in the gallbladder with fluke infection [5], SLE [6] or villous structures [7].

After hyperplastic polyps (HP) and sessile serrated lesions (SSL), traditional serrated adenoma (TSA) is the least frequent type of serrated lesions with a heterogenous molecular background. TSAs have three main characteristical features: 1) ectopic crypt formation/focus (ECF), 2) slit-like epithelial serration of the lumen, 3) tall columnar epithelial cells with eosinophylic cytoplasm. If the nuclei of these cells are centrally located and penicilliform it is called serrated senescence, but if nuclei become atypical, vesicular, crowded, it is regarded as serrated dysplasia. Of these three features, two should be present—with having greater than 50% proportion—for diagnosing a TSA [8, 9]. The importance of (colorectal) serrated lesions is increasing since these lesions are thought to be significant steps of the serrated pathway of colorectal carcinogenesis, which might be present in up to 30% of colorectal cancers [10]. While the presence of TSAs in the large bowel is widely accepted [9, 13], their occurence in the upper gastrointestinal (GI) tract or in extracolonic locations is just being recognized with several case studies [14–18] and a singlecase report of a serrated adenoma of the gallbladder [19]. In a series of Rubio et al. these upper GI-tract TSAs tended to have an agressive behaviour highlighting the importance of the serrated pathway also in the upper GI tract [14].

## Case Description

Our patient was a 67-year old female with paroxysmal atrial fibrillation, with good cardial function according to her regular cardiological follow up. Her medical history also included osteoporosis, cysts in her liver and kidneys and smoking. No malignant disease or clinically important hereditary condition was noted. She had been suffering from abdominal complaints suggestive for chronic cholecystitis for 18 months and came to the surgical ward of Bajcsy-Zsilinszky Hospital for an elective surgical intervention. Physical exmination, ultrasound and laboratory results suggested a polypoid lesion related to chronic inflammation and a gallstone in the cholecyst. The lesion was not suspicious for malignancy or an infiltrative process, thus laparoscopic cholecystectomy was performed.

**FIGURE 1:**
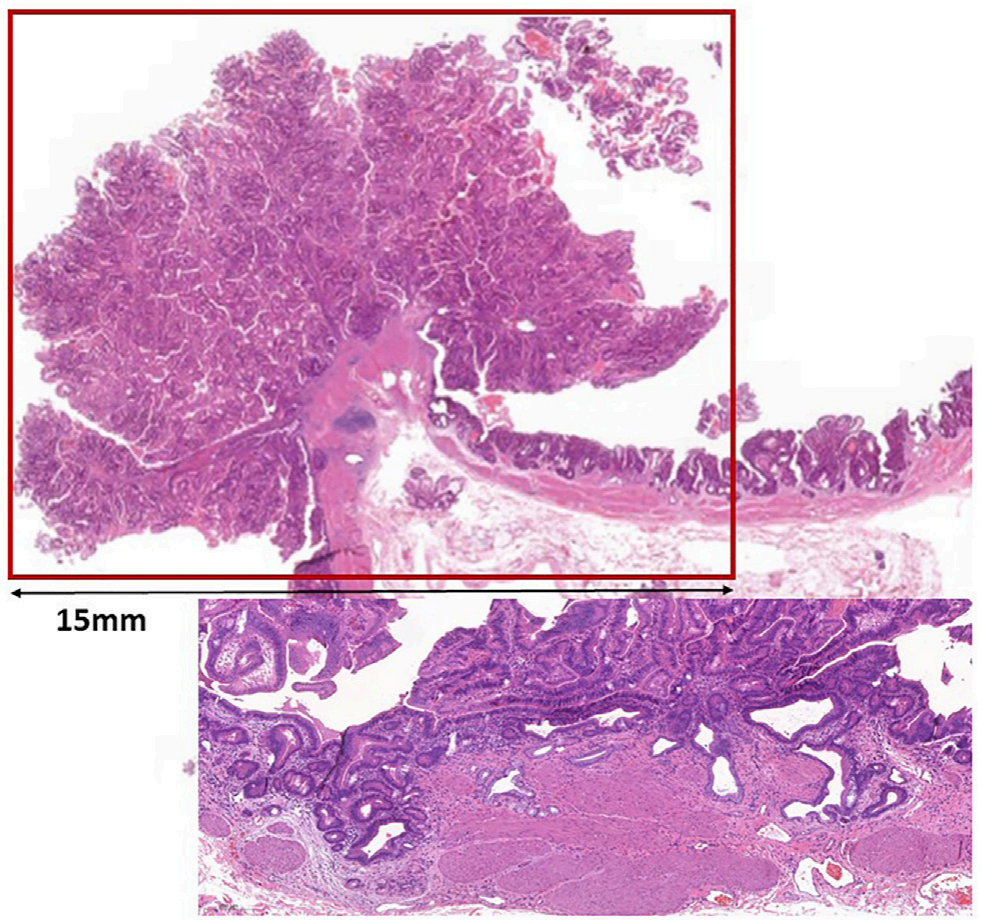
Low magnification picture of the polypoid mass protruding from the mucosa. Line shows the size of the lesion. Insert shows a focus with glands pushing lamina muscularis mucosae.

**FIGURE 2:**
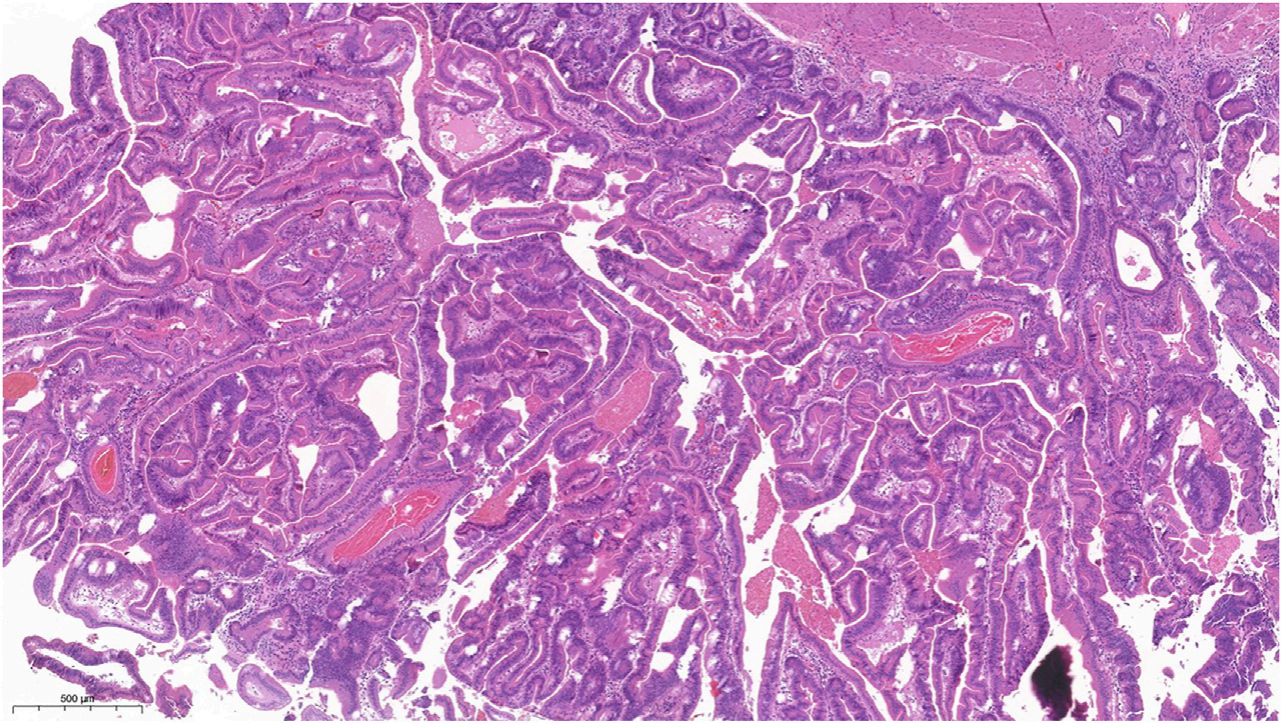
Picture of the complex structure with villiform adenomatous projections, lots of ectopic crypts and slit slike serrations. The epitheial cells showed mainly eosinophilic cytoplasm.

**FIGURE 3:**
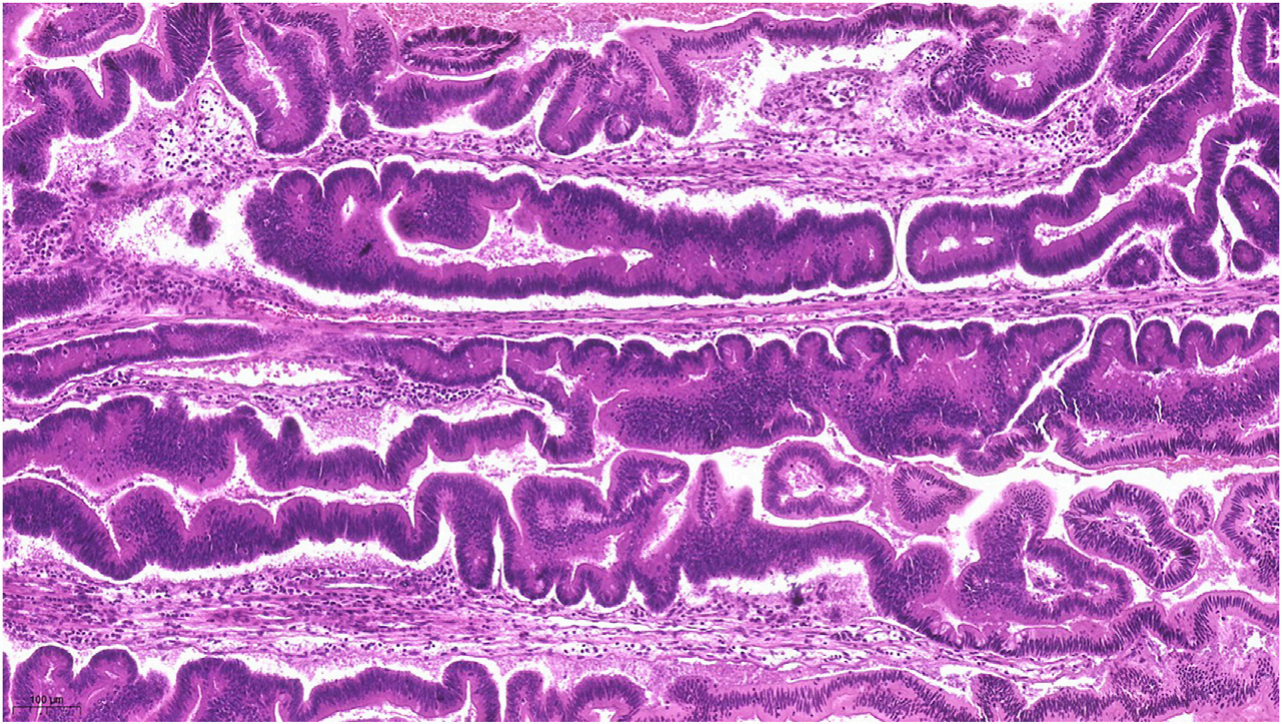
Represents the lots of ectopic crypt formation (ECF) showing an almost undulating pattern.

**FIGURE 4:**
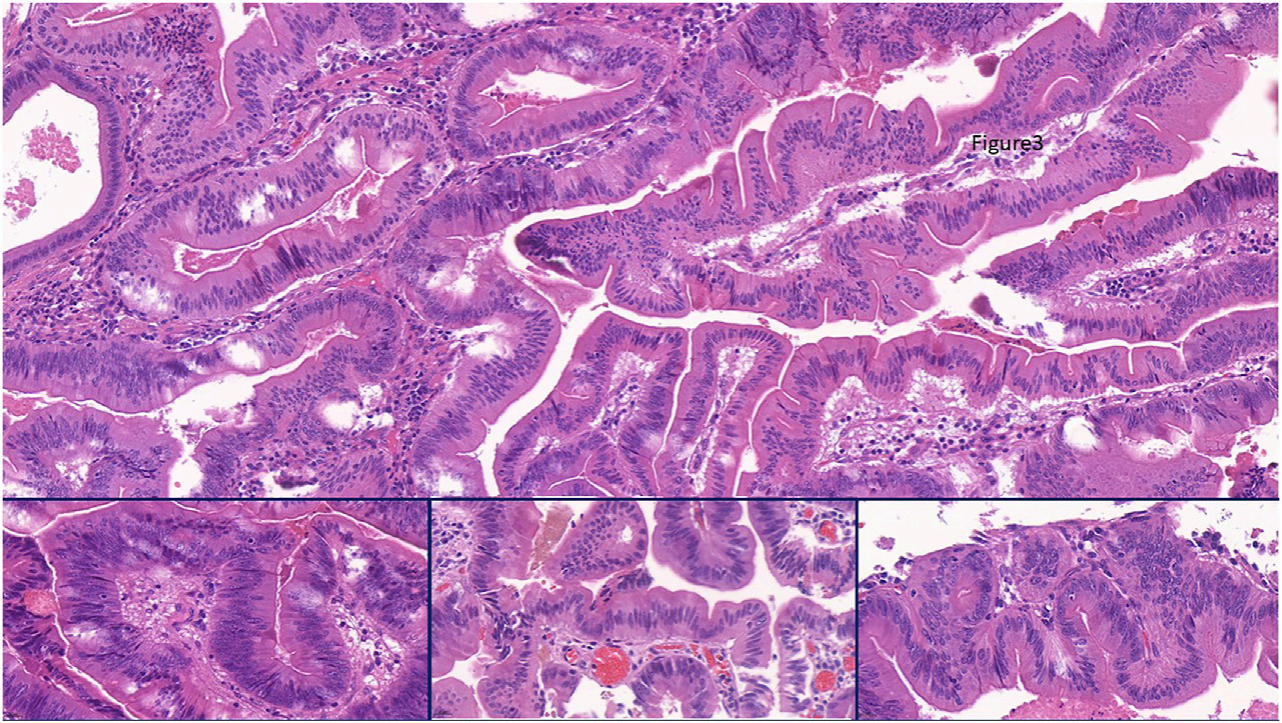
Representing the combination of slit like serrations and ectopic crypt formation (ECF) present throughout the lesion. Focally some stratification of nuclei appeared as sign of conventional dysplasia (Insert, left), but the columnar cells with bright eosinophilic cytoplam mainly had centrally located nuclei. Nuclear shape was partly of pennicilliform, corresponding to serrated senescence (insert, middle), but partly of roundish with open nuclear structure as a sign of serrated dysplasia (insert, right).

Macroscopically, a polypoid lesion with a diameter of 15 mm and with velvety surface emerged from the mucosa of the removed gallbladder. The polyp showed neoplastic appearance, however, did not seem to be infiltrative or malignant (Figure 1). The muscular layer was intact, only a single focus was found where few adenomatously transformed crypts protruded into the slightly fibrotic muscular layer, but this was not interpreted as true invasion (insert at Figure 1). Microscopically, the polypoid lesion showed thickening of the mucosa with complex structure. Some dilated and atrophic glands were found at the basal part of the specimen, but the polypoid mucosa merely consisted of complex, villous and papillary epithelial structures (Figure 2). Slit-like serrations and ectopic crypt foci were present throughout the lesion, latter partly formed undulating appearance (Figure 3). The epithelial cells mainly showed prominent eosinophilic cytoplasm with centrally located nuclei, which partly were of penicilliform shape suggesting serrated senescence. But on other foci vesicular nuclear structure also appeared as sign of serrated dysplasia, sometimes even reaching high-grade dysplasia. On certain areas, conventional dysplasia appeared with multilayered, stratified nuclei (Figure 4). Cribriform structures or invasion was not visible.

Immunohistochemical reactions with the four mismatch repair proteins (MMR) (MLH1, MSH2, MSH6, PMS2) showed retained nuclear staining, claiming for MMR-proficiency. *BRAF* was of wild type, but a *KRAS* G12D mutation, a frequent (∼50%) finding in TSAs, was identified [20].

## Follow Up and Outcome

As bile discharge was noted postoperatively through the surgical drains, the urgently performed ERCP found a stenotic main biliary duct and bile leakage from the bed of the gallbladder. This situation was resolved by the implantation of an endobiliary stent. The patient became asymptomatic and was discharged home soon. The 5-years follow up found the patient well, without any complications related to her TSA.

## Discussion

Our case was an unexpected finding in a routine cholecystectomy performed for chronic cholecystitis. Preoperative examinations suggested some polypoid structure growing in the gallbladder on the background of chronic inflammation, but no suspicion for malignancy. Macroscopic picture suggested also a benign polyp. In fact, the most important differential diagnostic question was to rule out a possible malignancy in the gallbladder, since this have a dismal prognosis. Gallbladder polypoid changes usually represent cholesterolosis, but in case of bigger lesions, an adenoma is more probable. Non-epithelial neoplastic lesions can also present as polypoid structures, but our case was clearly of epithelial origin.

Firstly, the tubulovillous structures and conventional dysplasia were suggestive of a tubulovillous adenoma (TVA). TVAs are rare in gallbladders, but do occur and follow the adenoma-carcinoma sequence known from colorectal carcinogenesis [21]. Further examination recognized complex papillary structures with the dominance of ECFs, eosinophilic cytoplasm, serrated features and slit-like serrations, therefore we diagnosed the case as a TSA of the gallbladder.

Ersöz et al. compared the imunohistochemical characteristics of the TVAs and the TSAs, finding that CK20 and CK7 were mainly expressed in ECFs, which also showed elevated ki-67 index. MUC5AC and cytokeratin seven coexpression was more frequent in TSAs. P16 stained more TSAs, while p53 highlighted the dysplastic compartments in both groups. But the authors also concluded that from the several histologic features shared by both TVAs and TSAs, the predminance of mid-zonal nuclei, eosinophilic cells, slit-like serrations and ECFs proved to be the most discriminatory features for TSAs [22].

Intracholecystic papillary neoplasm (IPN) of the gallbladder also emerged as differential diagnosis. The gastric/pyloric and intestinal types were easily excluded by their appearance. The biliary phenotype usually forms papillae lined by cuboidal cells with clear to eosinophilic cytoplasm and enlarged nuclei with distinct nucleoli. This usually harbours *KRAS* mutation and carries the highest risk for associated invasive carcinoma. Our lesion showed only eosinophilic cytoplasm, not clear cytoplasm. The cells were not cuboidal, but were more columnar, elongated, focally with sratification of the nuclei. The least frequent oncocytic phenotype of IPNs forms arborizing papillae lined by oncocytic cells with atypia. Our lesion did not show arborizing papillae, the nucleoli were not large or eccentric. Conventional and serrated senescence/dysplasia dominated the structure, which is not a usual feature of oncocytic papillary neoplasms. Neither the slit-like serration and lots of ectopic crypt foci present in our case are characteristic for any type of IPN. Cytokeratin 7, MUC1 and HepPar1 immunohistochemical reactions could help us to exclude these entities. As the picture (with the ECF, slit-like serrations and serrated senescence/dysplasia) was overwhelmingly suggestive of a TSA, we did not perform any additional immunohistochemical reactions.

For decades, the conventional adenoma-carcinoma sequence was considered as the main pathway of colorectal carcinogenesis, but the serrated lesions with the interval cancers have driven our attention towards the serrated pathway [23, 24]. HPs, SSLs and TSAs are part of this pathway, with TSAs being the least common, but well-known entity in the colon. TSAs as of now, are considered as high-risk precursors of colorectal cancers [12]. Colonic serrated lesions show strong co-occurence with other (pre)neoplastic lesions throughout the colon [20]. TSAs in the bowel are more prone to be accompanied by other precursor lesions [11] or high-risk adenomas [12] driving our attention of TSAs as index lesions showing higher risk of having neoplastic processes in the bowel. Serrated lesions usually have characteristic molecular background with activating mutations in the MAPK-pathway. While SSLs usually harbour *BRAF* mutations and trend to be MMR-deficient, the majority of TSAs are MMR-proficient and present with *KRAS* mutation (closely followed by *BRAF* mutations) [20]. There are insuffcient data available about the molecular features of extracolonic TSAs. Our case showed similar findings to colonic TSAs (*KRAS* mutation and MMR-proficiency), which strengthened our diagnosis and hypothetized similarities to colonic TSAs [9].

Recently TSAs were also described in the upper GI tract with cases in the esophagus [25], stomach [15, 17], duodenum [26], pancreas [16], small bowel [15] and a single case in the gallbladder [19], emphasizing the possible role of the serrated pathway in the upper or extracolonic GI tract. Furthermore, a comprehensive review of Rubio et al pointed out, that upper GI tract TSAs have special importance, since these were more likely to show invasive growth or accompanied by invasive cancer. In detail, 39 of the 73 reported TSAs (53.4%) showed invasive behaviour [14]. Growing interest drives our attention towards TSAs, although their extracolonic manifestation is rarely reported. Our case is a unique case with known molecular background, but more data on extracolonic TSAs are needed to better understand their occurence, importance and clinical relevance.

## Conclusion

Adenomas are infrequent findings in gallbladders and serrated lesions are exceptionally rare in this location with only a single serrated adenoma with a concomitant carcinoma reported until now. TSAs are the least frequent serrated lesions of the serrated pathway, which is involved in colorectal carcinogenesis in almost one third of CRCs and usually follow the KRAS/BRAF-mutated pathway. Until now, the relevance of the serrated pathway in extracolonic neoplasias is not well known, thus our case may add some information to this field.

## Take Away

Sometimes a least interesting specimen can bring huge surprise and serve as a unique case. We all know that common diagnoses are the most common ones, thought, we should not hesitate to diagnose something out-of-the box. Gallbladder specimens are usually handled with less interest, because of their usually uniform character. Still, we need to keep our eyes wide open towards morphological characteristics—even for other fields—to be able to find something extraordinary. To our knowledge, our case is among the pioneers of TSAs described in gallbladder.

## Data Availability

The raw data supporting the conclusion of this article will be made available by the authors, without undue reservation.

## Abbreviations

CRC, colorectal cancer; ECF, ectopic crypt formation/focus; ERCP, endoscopic retrograde cholangiopancreatography; GI, gastrointestinal; HP, hyperplastic polyp; MMR, MisMatch Repair; SSL, sessile serrated lesion; TSA, traditional serrated adenoma; TVA, tubuloVillous adenoma; SLE, systemic lupus erythomatosus.
